# Targeted DNA damage at individual telomeres disrupts their integrity and triggers cell death

**DOI:** 10.1093/nar/gkv598

**Published:** 2015-06-15

**Authors:** Luxi Sun, Rong Tan, Jianquan Xu, Justin LaFace, Ying Gao, Yanchun Xiao, Myriam Attar, Carola Neumann, Guo-Min Li, Bing Su, Yang Liu, Satoshi Nakajima, Arthur S. Levine, Li Lan

**Affiliations:** 1 School of Medicine, Tsinghua University, No. 1 Tsinghua Yuan, Haidian District, Beijing 100084, China; 2 University of Pittsburgh Cancer Institute; University of Pittsburgh School of Medicine; 5117 Centre Avenue, Pittsburgh, PA 15213, USA; 3 Department of Microbiology and Molecular Genetics; University of Pittsburgh School of Medicine; 523 Bridgeside Point II, 450 Technology Drive, Pittsburgh, PA 15219, USA; 4 Xiangya Hospital, Central South University, Changsha, 410000, China; 5 Departments of Medicine and Bioengineering, University of Pittsburgh, 3550 Terrace Street, 1218 Scaife Hall, Pittsburgh, PA 15261, USA; 6 Department of Pharmacology and Chemical Biology, University of Pittsburgh School of Medicine. W1340 Biomedical Science Tower 3, 200 Lothrop Street, Pittsburgh, PA 15213, USA; 7 Graduate Center for Toxicology, Markey Cancer Center, University of Kentucky College of Medicine, 1905 V.A. Drive, 306 Health Science Research Building, Lexington, KY 40536, USA; 8 Shanghai Institute of Immunology, Shanghai JiaoTong University School of Medicine, Shanghai, China; 9 Department of Immunobiology and Vascular Biology & Therapeutics Program, Yale University School of Medicine, New Haven, CT 06520, USA

## Abstract

Cellular DNA is organized into chromosomes and capped by a unique nucleoprotein structure, the telomere. Both oxidative stress and telomere shortening/dysfunction cause aging-related degenerative pathologies and increase cancer risk. However, a direct connection between oxidative damage to telomeric DNA, comprising <1% of the genome, and telomere dysfunction has not been established. By fusing the KillerRed chromophore with the telomere repeat binding factor 1, TRF1, we developed a novel approach to generate localized damage to telomere DNA and to monitor the real time damage response at the single telomere level. We found that DNA damage at long telomeres in U2OS cells is not repaired efficiently compared to DNA damage in non-telomeric regions of the same length in heterochromatin. Telomeric DNA damage shortens the average length of telomeres and leads to cell senescence in HeLa cells and cell death in HeLa, U2OS and IMR90 cells, when DNA damage at non-telomeric regions is undetectable. Telomere-specific damage induces chromosomal aberrations, including chromatid telomere loss and telomere associations, distinct from the damage induced by ionizing irradiation. Taken together, our results demonstrate that oxidative damage induces telomere dysfunction and underline the importance of maintaining telomere integrity upon oxidative damage.

## INTRODUCTION

Telomere DNA is characterized by the TTAGGG repeats seen at the ends of chromosomes. This repetitive DNA forms T-loops, a D-loop, and G-quadruplex structures (1) and is capped by the telomere shelterin protein complex, including telomere repeat binding factor 1 (TRF1), TRF2, TIN2, TPP1, POT1 and RAP1. Among these proteins, TRF1 directly binds duplex TTAGGG repeats and specifically localizes to telomeres (2,3). Studies in a variety of human diseases, both inherited and acquired, yield ample evidence that telomere dysfunction is a key driver of aging-related degenerative pathologies and increased cancer risk. The telomeres of different chromosomes may have different impacts with respect to cell biology and disease. Therefore, given the 92 telomeres in human cells, identifying the impact of DNA damage at individual telomeres would be potentially useful in exploring telomere biology and oncogenesis (4–6). Oxidative stress seems to contribute to telomere shortening that is particularly significant at the incomplete ends of replicated chromosomes (7). Stress-induced damage is mainly caused by reactive oxygen species (ROS) that are generated endogenously during cellular respiration or exogenously during infection or exposure to chemical and physical agents (8). Although the effect of telomere oxidative DNA damage has been investigated by exposing cells globally to oxidants, chemicals or radiation, the main challenge is that these conventional approaches also induce damage throughout the whole genome, whereby a large amount of genomic damage, alterations of gene expression and mitochondrial dysfunction occur that indirectly affects telomeres (9–11). As a result, it is not clear whether the observed cellular responses are due to damage of the entire genome or the impact of damage on telomeres. Thus, whether oxidative stress-induced telomere damage could be directly and singularly responsible for telomere shortening and dysfunction remains unresolved.

To address this question, we developed a novel method termed KR-TEL (KillerRed induced DNA damage at telomeres). KillerRed (KR) is a unique fluorescent protein capable of generating site-specific ROS upon visible light illumination (550–580 nm) (12–14). We fused the KR encoding sequence to the TRF1 sequence, resulting in a chimeric protein, KR-TRF1, that introduces oxidative DNA damage specifically at the sites of telomeres. In this report, we present evidence that telomeric damage induces cell senescence and cell death without the major confounding effects of oxidative stress elsewhere in the cell. We found that telomeric oxidative DNA damage is a potent inducer of telomere shortening. Our results also revealed two major types of chromosomal aberration, chromatid telomere loss and telomere associations, which may contribute to the cytogenetic signature of telomere DNA oxidative damage.

## MATERIALS AND METHODS

### Cell lines and transfections

U2OS, HeLa, MCF7, IMR90, MCF7 and BJ fibroblast cells were used in this study. All cell lines were cultured in Dulbecco's modified Eagle's medium (DMEM, Lonza) with 10% fetal bovine sera (Atlanta Biologicals) at 37°C and 5% CO_2_. KR-TRF1 and DsR-TRF1 expressing HeLa cell lines or IMR90 cells were established by infection with pLVX-IRES-Puro KR-TRF1 and DsR-TRF1 lentivirus respectively, and HeLa cells were selected with 1 μg/ml Puromycin (Hyclone). Plasmids were transfected with PolyJet (SignaGen) or Electroporation (NEPAGENE, NEPA21, 2 mm gap cuvettes) using a portion pulse of 150V, 5 msec at 50 msec intervals, two pulses and 10% decay rate and a transfer pulse of 20 V, 50 msec at 50 msec intervals, five pulses and a 40% decay rate (for U2OS cells).

### Plasmids

pEGFP-NTH1, FEN1 and polymerase β have been described (15). FLAG-TRF1-fok1 was used as described in a previous study (16). KR and DsRed DNA with additional Age I and EcoRI sites were amplified by polymerase chain reaction (PCR) and sub-cloned into a pYFP (Clontech) tagged TRF1 plasmid to generate pCMV KR-TRF1 and DsRed-TRF1 plasmids. KR-TRF1 and DsRed-TRF1 fragments were digested by KpnI and SmaI and sub-cloned into the KpnI–EcoRV sites of pcDNA5/FRT/TO (Invitrogen), respectively. pLVX-IRES-Puro KR-TRF1 and DsRed-TRF1 were made by PCR of KR-TRF1 or DsRed-TRF1 with additional SpeI and BamHI sites and sub-cloned into SpeI and BamHI sites of the pLVX-IRES-Puro (Clontech) vector. All PCR products were confirmed to have correct sequences. pSLQ1658-dCas9-EGFP and pSLQ1651-sgTelomere (F + E) were obtained from Addgene. pCMV-KR-TRF2 was made by PCR of KR with an additional AgeI and an XhoI site using 5’-AAACCGGTATGGGTTCAGAGGGCGGCCCCGCCCTG-3’ and 5’-CCGCTCGAGAGA TCTCGTCGTGGCTACCGATGGC-3’ as forward and reverse primers, respectively, and sub-cloned into the AgeI and XhoI sites of the pCMV-EYFP-TRF2 vector.

### Confocal microscopy

The Olympus FV1000 confocal microscopy system (Cat. F10PRDMYR-1, Olympus) with an FV1000 SIM Scanner and 405 nm laser diode (Cat. F10OSIM405, Olympus) was employed. FV1000 software was used for acquisition of images. For inducing DNA damage, a 405 nm laser was used with the indicated power; the output power of the 405 laser passed through the lens is 5 mW/scan. Laser light was passed through a PLAPON 60x oil immersion objective lens (super chromatic abe. corr. obj W/1.4NA FV, Cat. FM1-U2B990). Cells were incubated at 37°C on a thermo-plate (MATS-U52RA26 for IX81/71/51/70/50; metal insert, HQ control, Cat. OTH-I0126) in Opti-MEM during observation to avoid pH changes. For bleaching KR, a 559 nm laser was used. For counting foci positive cells, cells containing >5 colocalized foci with KR-TRF1 were counted. For calculation of the percentage of colocalization with KR-TRF1, foci positive cells in 50 cells were counted in every experiment. Three independent experiments were performed and representative data are shown. Fluoview Soft (Olympus) was used for data analysis. For quantification of the intensity of the damage response of proteins, a ratio of enrichment of the same area in a single cell nucleus was used. Here, mean intensity of accumulated proteins at the sites of KR-TRF1/mean intensity of proteins remote from the KR-TRF1 spot (background) in the same nucleus was calculated. A total of 50 spots in 10 cells were calculated. The +/− SD calculated in each case is shown in the Figure Legend. The *P*-value is calculated by Student's t-test using Stat Plus software; *P* < 0.005 is shown as **.

### STORM image

The commercial STORM microscope system from Nikon Instruments (NSTORM) was employed for STORM imaging. The cells grown on a glass-bottomed petri dish were fixed with 3.7% (v/v) formaldehyde for 15 min at RT, followed by standard immunofluorescence staining. Two color staining for STORM imaging was carried out by Alexa 488/647 F(ab’)2-goat anti-mouse/rabbit IgG (H + L) secondary antibody (catalogue #: A-11017 and A-21246). Immediately before imaging, the buffer was switched to the STORM imaging buffer according to the Nikon N-STORM protocol (50 mM Tris–HCl pH 8.0, 10 mM NaCl, 0.1M cysteamine (MEA), 10% w/v glucose, 0.56 mg/mL glucose oxidase, 0.17 mg/ml catalase). Images were reconstructed using a custom-written algorithm on Matlab. TetraSpeck microspheres (0.1 μm diameter, blue/green/orange/dark red fluorescence) were used for the chromatic correction for two color STORM images. For STORM, Cysteamine (MEA), Glucose Oxidase and Catalase were purchased from Sigma-Aldrich. TetraSpeck microspheres (0.1 μm diameter, blue/green/orange/dark red fluorescence) were purchased from Life Technologies.

### Three-dimensional structured illumination microscopic (3D-SIM) imaging

A 3D-SIM microscopy system (N-SIM, Nikon) was employed to carry out the 3D super-resolution imaging of U2OS cells. The wavelengths of 405 and 561 nm were used to excite DAPI and KR-TRF1 in the cell nucleus, respectively. An oil immersion objective (Nikon Apo TIRF 100x, NA = 1.49) was used for all 3D-SIM imaging. The lateral resolution of the 3D-SIM image is ∼100 nm and the axial resolution is ∼240 nm. The cells grown on a glass-bottomed petri dish were fixed with 3.7% (v/v) formaldehyde for 15 min at RT, followed by three washes with phosphate buffered saline (PBS) and then freshly made gelvatol was used as a mounting medium to cover the cells.

### KR activation

KR activation was conducted in two ways. Activation of KR in a single cell was performed with a 559 nm laser for 20 scans (1 mW/scan) only for the selected cell nucleus. Local activation of one KR spot was performed with the same 559 nm laser in a selected area within a single cell nucleus. One scan takes <1 s. Activation of KR in bulk cells was done by exposing cells to a 15 W Sylvania cool white fluorescent bulb for the indicated time (20 min to 4 h) in a stage UVP (Uvland, CA, USA). The dose of 559 nm laser light that was delivered to the KillerRed spot has been calculated previously (17). The KR-TRF1 (around 1 μm^2^ in diameter) spot is around 12 mJ/μm^2^. In the case of fluorescent light activation, the rate of light is 15 J/m^2^/s. With a 20 min −1 h light exposure, the final power delivered to each KR-TRF1 spot is around 20 mJ/μm^2^ - 60 mJ/μm^2^. Cells were placed under a water bottle (height to light is 15 cm) to prevent an increase of temperature during light activation.

### Immuno-assays and antibodies

For immunofluorescence staining, cells were fixed with 3.7% (v/v) formaldehyde for 15 min at RT, followed by three washes with PBS. Cells were then permeabilized with 0.2% Triton X-100 for 5 min at RT and washed with PBS twice. Primary antibodies were diluted in DMEM + Azide and incubated overnight at 4° and cells were washed three times with PBS and incubated with secondary antibodies diluted in DMEM + Azide for 30 min at RT. Cell samples were then mounted in drops of PermaFluor (Immunon). Primary antibodies used in this research were: anti-KR (1:200, Ab961, Evrogen), anti-DsR (1:100, abcam ab62341), anti-8-oxoG (1:100, Millipore MAB3569), anti-γH2AX (1:400, Millipore 05636), anti-polyADP-ribose (1:100, Millipore MAB3192) and anti-DMPO (1:100, abcam ab104902). Alexa Fluor 405/488/594 goat anti-mouse/rabbit immunoglobulin G or IgM (Invitrogen) was used. Sodium dodecyl sulphate-polyacrylamide gel electrophoresis and WB were described in previous studies (18). Anti-KR (Ab961, Evrogen), anti-TRF1 (santa cluz, sc-56807) and anti-actin (Calbiochem, CP01) were used.

### Cell proliferation and colony formation assays

U2OS cells were transfected with KR-TRF1 and DsR-TRF1 by electroporation. Twenty-four hours after transfection, 1 × 10^4^ cells were seeded into a 60 mm petri dish and the transfection efficacy was over 95%. The cells were treated with or without 15 W white light for 2 h and cells were then counted with a CASY cell counter (Roche) every 24 h for 5 days. For measuring cell survival by the colony formation assay using U2OS or HeLa cell lines, 350 cells were seeded on a 60 mm petri dish 24 h before light activation. Cells were treated with or without 15 W white fluorescent light for the indicated time period in PBS. Cells were removed with PBS and DMEM was added after treatment. After 10 days of culturing in the dark, cells were fixed and stained with 3.7% crystal violet in Methanol. Colonies were counted and calculated.

### MTT assay

IMR90 cells that stably expressed KR-TRF1 and DsR-TRF1, respectively, were seeded at a density of 5 × 10^3^ cells per well in 96-well plates 24 h before treatment at the indicated time of light exposure. Cell viability was determined 48 h after light activation with the MTT assay Kit (Promega). Absorbance was measured at 490 nm on a 96-well plate reader (VERSAmax tunable microplate reader, Molecular Devices). Results are presented as percentage of survival, with the control (untreated cells) as 100% survival.

### Telomere-PNA FISH analysis/telomeric quantitative FISH

Telomere-PNA FISH was performed using Telomere PNA FISH Kit/Cy3 (Dako) according to the provided protocol. Briefly, slides with chromosome spreads were fixed with 3.7% paraformaldehyde diluted in 1× Tris buffered Saline (TBS) for 2 min, washed 2× with 1× TBS, treated with pre-treatment solution and dehydrated with a cold graded ethanol series (70, 85 and 95%). After being air-dried, slides were incubated with 0.5 mg/ml Telomere PNA Probe/Cy3 for 2 h at RT. Following incubation, slides were washed in washing solution, dehydrated again with a cold graded ethanol series and mounted with mounting media including DAPI (Dako) prior to microscopy.

### Metaphase chromosome spreads

KR-TRF1 or DsR-TRF1 stably expressing HeLa cell lines were treated with 15 W white light for 1 h and subsequently incubated for 12 h. Cells were then arrested at metaphase with 0.1 μg/ml colcemid (Sigma) for 3 h. Cells were harvested by gentle pipetting, washed once in 1× PBS and incubated in 0.075M KCl at RT for 30 min. Following incubation, cells were fixed in fixative (3:1 methanol/glacial acidic acid) for 10 min and centrifuged and fixation repeated 3×. Cells were dropped onto wet slides and air-dried overnight in preparation for Telomere-PNA FISH analysis.

### Telomere length assay

KR-TRF1 and DsR-TRF1 stably expressing HeLa cell lines were used in this study. Cells were cultured for 30 passages and exposed to white fluorescent light for 30 min at each passage. The total genomic DNA was purified using the GeneJET Genomic DNA Purification Kit (Thermo Scientific). Telomere restriction fragment analysis was performed using the TeloTAGGG Telomere Length Assay Kit (Roche) according to the manufacturer's protocol. Briefly, 2.5 μg genomic DNA was digested with RsaI and HifI restriction enzymes, separated on a 0.8% agarose gel and transferred to a nylon membrane. Transferred DNA was fixed on the membrane by UV-crosslinking (120 mJ) and hybridized with a DIG-labeled telomere probe, followed by incubation with anti-DIG-alkaline phosphatase and detection by chemiluminescence.

## RESULTS

### Real time production of oxidative DNA damage with KR-TRF1 and light exposure

**Figure 1.**
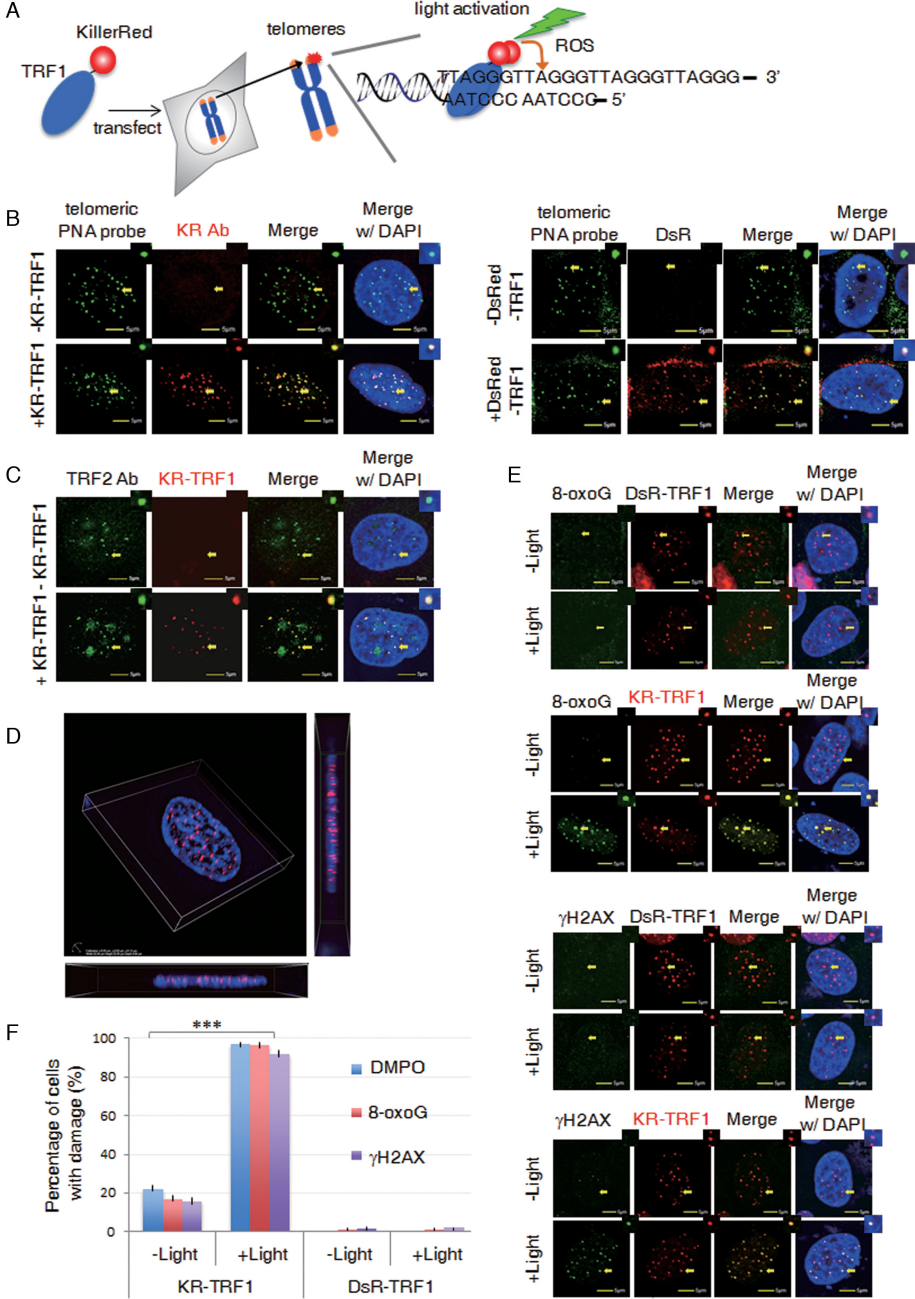
DNA Damage Targeted at Telomeres (DTT) using KillerRed-TRF1. (**A**) Schematic of the DTT system: visible light activation of KR-TRF1 induces localized oxidative DNA damage at telomere repeat sequences in living cells. (**B**) Colocalization of KR-TRF1 and PNA FISH-labeled telomeres in U2OS cells with or without transfection of KR-TRF1 (*left panel*) or DsRed tagged TRF1 (DsR-TRF1) detected by KR or DsRed antibody (*right panel*). (**C**) Immuno-staining of endogenous TRF2 with or without KR-TRF1 expression in U2OS cells. (**D**) Representative three-dimensional super-resolution image of KR-TRF1 using 3D-SIM. The lateral resolution is ∼100 nm and the axial resolution is ∼240 nm. (**E**) Production of telomeric site-specific DNA damage by KR-TRF1 after light illumination. Immuno-staining of DsR-TRF1 or KR-TRF1 transfected U2OS cells with anti-8-oxoG and anti-γH2AX. Scale bar equals 5 μm. (**F**) Quantification of colocalizations from (e). Mean values with SDs from 150 cells in three independent experiments are given. *P*-value is calculated by Student's *t*-test, ** *P* < 0.005. Arrows indicate the enlarged area shown at the right top of each image.

The ability to connect oxidative base damage directly with telomere shortening and telomere dysfunction-induced senescence has been hampered by the lack of an effective methodology to confine the damage specifically to the telomeres. To overcome this obstacle, we developed the KR-TEL (KillerRed induced DNA damage at telomeres) method using a KR-TRF1 fusion to deliver localized oxidative DNA damage. KR can be activated to produce reactive oxygen with visible light or 559 nm laser light using confocal microscopy in real time (Figure 1a). To rule out the possibility that ectopically expressing KR-TRF1 may impact telomere function (19), we constructed a non-phototoxic red fluorescent protein (DsRed)-tagged TRF1 (DsR-TRF1) as a functional control for ectopic expression of KR-TRF1. To analyze the functional impact of location-defined telomeric oxidative damage in different cell types and telomere regeneration backgrounds, we used a U2OS cell line with the alternative lengthening (ALT) pathway to elongate telomere length, HeLa and 293 cell lines expressing active telomerase, and a human diploid fibroblast IMR90 cell line without active telomerase (20–22). We verified the specificity of KR-TRF1 and DsR-TRF1 in telomere targeting, we performed telomere-specific FISH using telomeric peptide-nucleic acid (PNA) probes in U2OS cells. Both proteins showed precise co-localization with telomeric PNA probes (Figure 1b). Endogenous telomere repeat binding factor 2 (TRF2), another shelterin complex component that binds to telomere repeats, also colocalized with KR-TRF1 in U2OS cells (Figure 1c), confirming that expression of TRF1 fusion proteins does not interrupt the binding of TRF2 to telomeric DNA repeats. The binding of TRF2 to telomeres appeared not as specific as TRF1, because TRF2 also exhibits higher expression in the nucleolus (Figure 1c) as has been shown previously (23). Super-resolution imaging of KR-TRF1 expression was acquired with three-dimensional structured illumination microscopy (3D-SIM) (Figure 1d, Supplementary Video), confirming that telomeres form distinct spots in three-dimensions at the super-resolution level. Recently, a telomere visualization CRISPR system was developed to enable imaging of endogenous telomeres by a small guide RNA for telomere sequences (sgTelomere) and dCas9-EGFP, which does not have nicking activity (24). We expressed dCas9-EGFP and sgTelomere^(F + E)^ together with KR-TRF1 in U2OS cells and IMR90 cells. Co-localization of KR-TRF1 with dCas9-EGFP at telomeres was observed in both cell lines (Supplementary Figure S1a), reflecting the specificity of KR-TRF1 at telomeric sequences. Together, these data demonstrate that KR-TRF1 behaves similarly as TRF1 at the telomere level.

The expression of KR-TRF1 in both primary and cancer cell lines shown in Supplementary Figure S1b, including human primary cells (BJ fibroblasts and IMR90 cells) and cancer cells (MCF7, U2OS and HeLa). To rule out the effects of TRF1 and KR overexpression, DsR-TRF1 was used as controls in the studies described below. DsR-TRF1 and KR-TRF1 levels in whole cell lysates are nearly equal in U2OS cells (Supplementary Figure S1c). Stable expression at a similar level of DsR-TRF1 and KR-TRF1 in HeLa cells is shown by western blot (WB) in Supplementary Figure S1d. Therefore, the potential effects induced by KR-TRF1 are not due to the differential expression of KR-TRF1 compared to DsR-TRF1.

Next, we confirmed damage production by KR at telomeres. We have shown that localized KR and controlled light activation yield spatially limited production of superoxide only in the immediate proximity (17). To validate telomere-specific damage production by KR-TRF1, we analyzed 8-oxo-Guanine (8-oxoG), a major lesion induced by oxidative stress both in U2OS (Figure 1e) and HeLa cells (Supplementary Figure S1e), and γH2AX, a surrogate marker of DNA double-strand breaks (DSBs) (17). Both markers were detected at sites of KR-TRF1 upon light activation (Figure 1e). Activation of KR-induced damage at telomeres dramatically increased the expression of DNA damage markers at the telomeres from a basal level of ∼10% to over 90%. As a control, DsR-TRF1, expressed at a similar level as KR-TRF1, yields only a basal level of DNA damage markers before or after light illumination (Figure 1f). These data indicate that oxidative DNA damage is specifically and efficiently induced at sites of telomeres after KR activation.

### Repair of 8-oxoG at individual telomeres in U2OS cells

**Figure 2.**
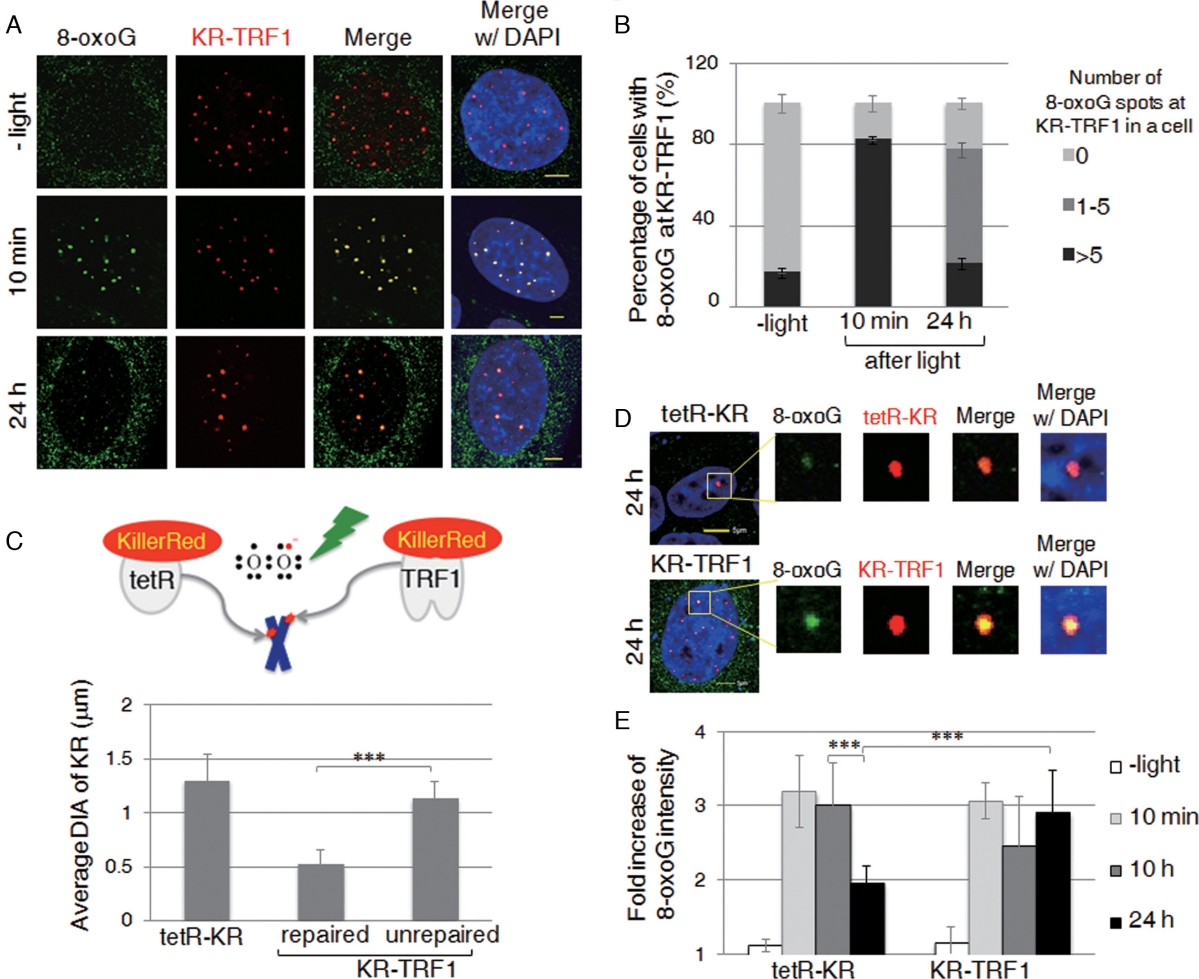
Long telomeric repeats have less efficient DNA repair compared to chromosomal DNA with a similar amount of DNA damage. (**A**) Staining of 8-oxoG in KR-TRF1-expressing U2OS cells with or without light activation. Cells were collected after recovery in the dark for the indicated times. Scale bar equals 5 μm. (**B**) Percentage of cells showing co-localization of 8-oxoG with KR-TRF1 spots, respectively. Each data point was derived from >150 cells in three independent experiments. Error bars depict standard deviations. (**C**) Schematic for generating single genome locus damage using tetR-KR and using KR-TRF1 to target damage to telomeres. Quantification of average diameters (DIA) of tetR-KR and KR-TRF1 spots with or without co-localization with 8-oxoG in KR-TRF1-expressing U2OS cells after 24 h recovery in the dark (*n* = 10). (**D**) Kinetics of 8-oxoG at sites of tetR-KR or KR-TRF1 transfected U2OS (TRE) cells with or without light activation of KR, recovered in the dark for the indicated time. (**E**) Quantification of the intensity of 8-oxoG at sites of tetR-KR or KR-TRF1 (*n* = 10) at the indicated time point after light activation of KR for 20 min.

Exposure to exogenous DNA damaging agents stresses numerous cellular functions, including gene expression and mitochondrial metabolism, which can indirectly impact telomeres (9–11). Therefore, telomere-specific damage formation allows an accurate assessment of the damage repair process related to telomeres. After inducing damage specifically at telomeres, we monitored the repair of 8-oxoG DNA damage by measuring the number and intensity of 8-oxoG-positive staining spots colocalized with KR-TRF1 after light illumination. We observed that 90% of cells showed 10–20 spots of KR-TRF1 colocalizing with 8-oxoG 10 min after light illumination (Figure 2a). After post-light recovery in the dark for 24 h, the percentage of cells with over five 8-oxoG-KR-TRF1 colocalizing spots returned to a near background level (Figure 2b), indicating that 8-oxoG has been removed to a large extent at the sites of telomeres. Interestingly, we found that approximately 50% of cells retained 1–4 spots of colocalization between 8-oxoG and KR-TRF1 that were not observed in the control (Figure 2b) after post-light recovery in the dark for 24 hr. This result indicates that 8-oxoG damage removal is not completely and uniformly removed among all telomeres.

### Long telomeric repeats have less efficient DNA repair compared to chromosomal DNA with a similar amount of DNA damage

The observation that a subpopulation of telomeres exhibits less efficient damage repair prompted us to identify the potential cause of the incomplete damage removal. To this end, we compared the size of KR-TRF1 colocalized with repaired or unrepaired foci and found that unrepaired 8-oxoG preferentially colocalized with relatively large KR-TRF1 foci. The average diameter (DIA) of KR-TRF1 foci at unrepaired telomeres (∼1 μm) is significantly larger than those at repaired telomeres (∼0.5 μm) (Figure 2c). It has been established that the heterogeneous lengths of telomeres can be reflected in the intensity of telomeric PNA staining (24). This is consistent with the KR-TRF1 staining in the U2OS cells in our experiments. Most likely, the amount of oxidative DNA damage introduced at KR-TRF1 binding sites is proportional to the telomere length upon light illumination. To compare the repair efficiency at telomeric sites versus non-telomeric sites, we employed tet-repressor (tetR)-KR that targets tetracycline repressive elements (TREs) integrated at a defined chromosomal locus (17,25) in U2OS cells to induce localized DNA damage within heterochromatin. As illustrated in Figure 2c, 200 copies of TRE were integrated at a defined genomic locus in U2OS TRE cells. As shown in previous publications, DAPI staining in the FISH assay (showing increased intensity) and immunofluorescence (IF) of heterochromatin markers (17,25) indicate that integrated repeat sequences form heterochromatin structures. The size of KR-TRF1 at unrepaired telomeres is nearly equal (∼1 μm diameter in size) to that of tetR-KR in heterochromatin (Figure 2c). Since telomeres coated by shelterin proteins form heterochromatin structures (26), we therefore assume that the damage production is equivalent for KR-TRF1 (∼1 μm DIA) and tetR-KR. Previously, we measured tetR-KR-dependent 8-oxoG intensity and correlated this with exogenous hydrogen peroxide (H_2_O_2_) treatment as a quantification parameter for damage introduction (17). The intensity of 8-oxoG at sites of KR-TRF1 (∼1 μm) equals treatment with 1–1.5 μM H_2_O_2_ after calculation, which is comparable to the damage production by tetR-KR, supporting the conclusion that equal amounts of damage are initially produced at both KR-TRF1 (∼1 μm DIA) and tetR-KR sites upon light activation. We then monitored 8-oxoG foci that colocalized with the same diameter in size (∼1 μm) for both KR-TRF1 and tetR-KR (Figure 2d). The intensity of 8-oxoG signals significantly decreased at sites of tetR-KR 24 hr after light illumination, while 8-oxoG at KR-TRF1 sites remained unchanged (Figure 2d and e). This result indicates that the repair of 8-oxoG at telomeres containing long telomeric repeats is less efficient compared to the same amount of damage located in chromosomal DNA in heterochromatin. We further determined the repair rate of 8-oxoG in HeLa cells (Supplementary Figure S1f). We observed that up to 50% of cells showed 10–20 spots of KR-TRF1 colocalizing with 8-oxoG 10 min after light illumination, which is less than that in U2OS cells. Importantly, in contrast to U2OS cells, the trend of repair in HeLa cells after post-light recovery in the dark for 24 h returned to a background level without showing an enhanced portion of unrepaired 1–4 8-oxoG foci at KR-TRF1 sites. This result indicates that the damage repair process occurs in HeLa cells as well as in U2OS cells. Since HeLa cells have homogenous telomeres, this result suggests that the heterogeneous removal of 8-oxoG in U2OS cells might be caused by the heterogeneity of their telomeres.

**Figure 3.**
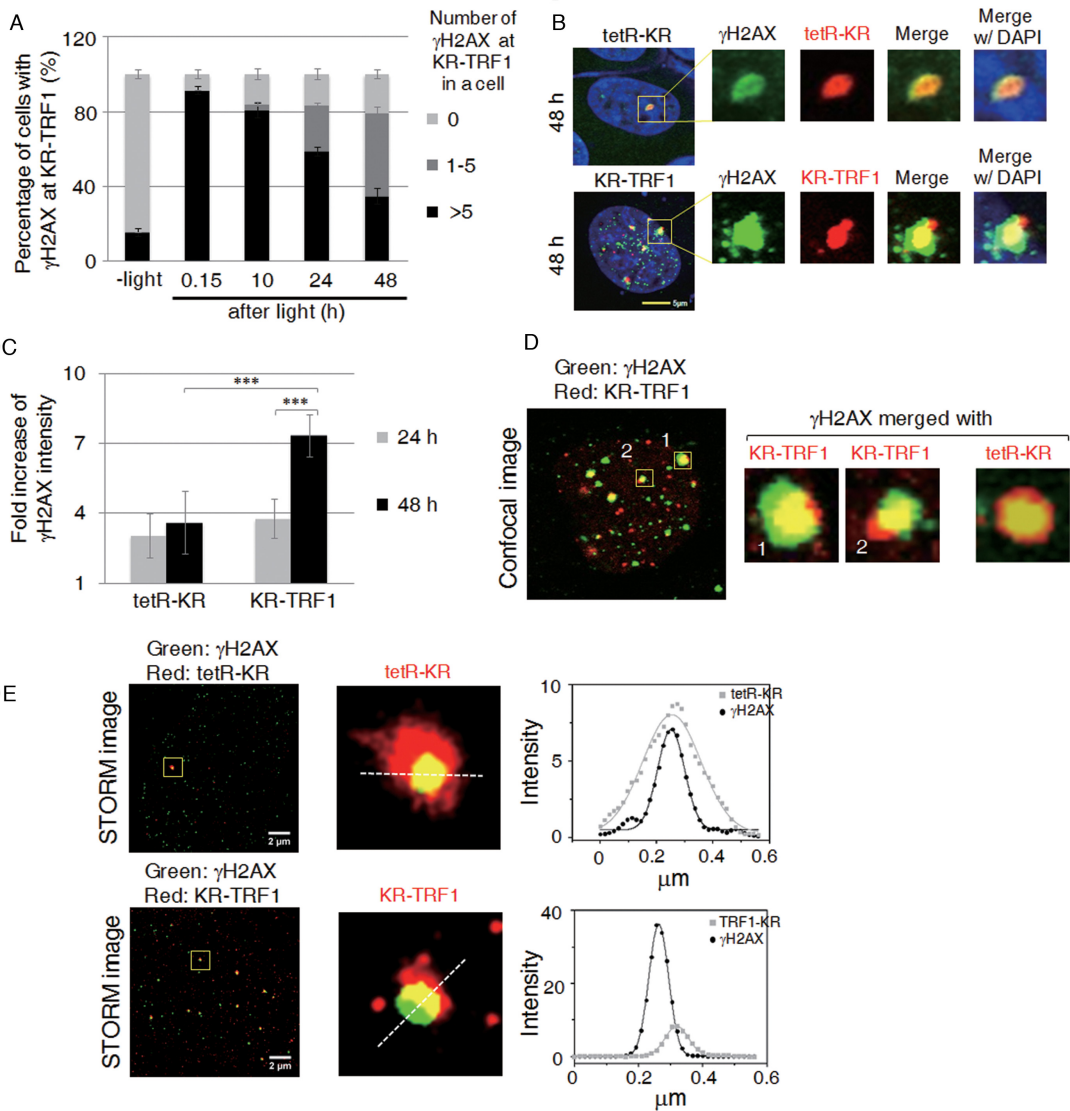
Expansion of γH2AX in long telomeric repeats. (**A**) Quantification of co-localization between γH2AX and KR-TRF1 signals. Error bars are derived from SDs from three independent experiments counting 150 cells each time. (**B**) Immunostaining of γH2AX at sites of tetR-KR- or KR-TRF1-expressing U2OS cells with or without light activation of KR after recovery in the dark for 24 h. (**C**) Quantification of the γH2AX signals at the sites of tetR-KR or KR-TRF1 (*n* = 10) 48 h after 20 min light activation. (**D**) Enlarged images of γH2AX at sites of KR-TRF1 or tetR-KR 48 h after 20 min light activation. (**E**) STORM images of **γ**H2AX at sites of KR-TRF1 or tetR-KR 48 h after light activation. Yellow box indicates region shown in middle panel. Left panel, linearized intensity analysis of **γ**H2AX and KR-TRF1 or Tet-KR. White dashed line denotes the path whereby the intensity was measured.

In parallel, we analyzed the kinetics of the γH2AX signal as a function of 8-oxoG repair. Similar to 8-oxoG signals, a decrease of γH2AX foci (90 to 38%) at KR-TRF1 sites is seen 24 h after light activation (Figure 3a, black bar). Consistent with the result with 8-oxoG foci, around 50% of cells retained less than five γH2AX foci (Figure 3a gray bar), indicating a repair-attenuated telomere sub population (Figure 3b). In contrast to 8-oxoG, the intensity of γH2AX did not decrease at sites of tetR-KR. Moreover, the intensity of γH2AX foci at sites of KR-TRF1 at 48 h increased approximately two-fold compared to that at 24 h (Figure 3c). The increased intensity of γH2AX might be caused by expansion of γH2AX through secondary damage such as conversion to DSBs via DNA replication. Enlarged images show that γH2AX foci expanded unidirectionally at KR-TRF1 sites, suggesting that the expansion is from the ends of chromosomes, i.e. the damage is induced at telomeres (Figure 3d, left). This phenomenon was not observed with tetR-KR, in which γH2AX is detected as a bidirectional expansion of staining located in the center of the non-telomeric area (Figure 3d, right). To determine the distribution of γH2AX under super-resolution, we utilized stochastic optical reconstruction microscopy (STORM) in this study to analyze the ultrastructure of γH2AX at damage sites, in addition to using a conventional confocal microscope. As observed under confocal microscopy, γH2AX staining shows unidirectional expansion at sites of KR-TRF1 in these super-resolution images and in a quantified graph measuring the center of distribution of foci (Figure 3e). Together, the comparison of individual telomeres and the non-telomeric region indicates that DNA damage is not repaired efficiently at sites that have long telomeric repeats in U2OS cells.

### Visualization of the DNA damage response at sites of damaged telomeres

**Figure 4.**
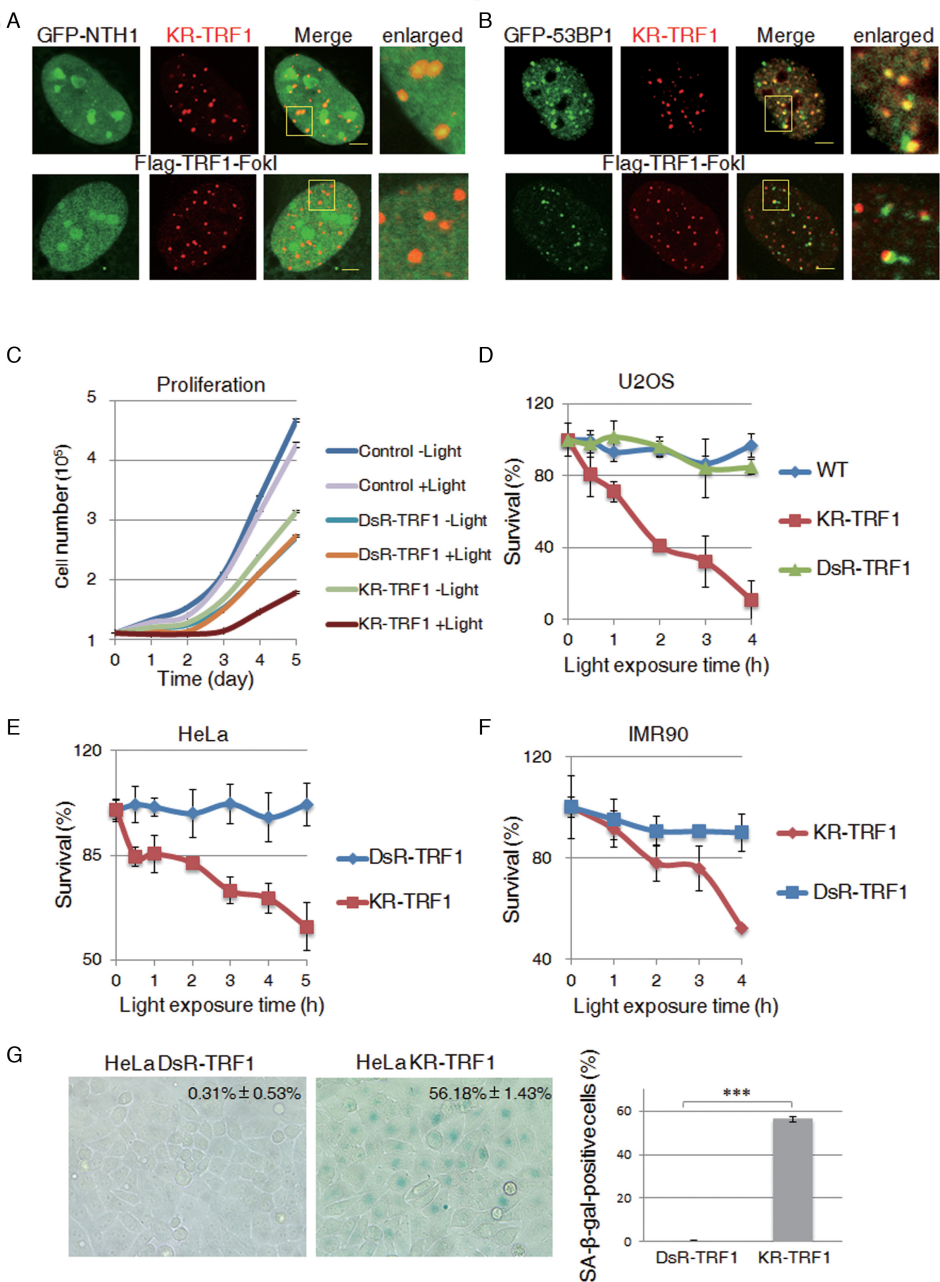
Oxidative DNA damage at telomeres leads to cellular senescence. (**A**) NTH1 is recruited to sites of KR-TRF1 induced damage but not to Flag-TRF1-FokI induced damage. KR-TRF1 expressing U2OS cells were light activated for 20 min and then fixed immediately for imaging. For Flag-TRF1-fokI analysis, U2OS cells were fixed 24 h after transfection with Flag-TRF1-FokI. Scale bar equals 5 μm. (**B**) The recruitment of 53BP1 to damaged telomere sites induced by KR-TRF1 and Flag-TRF1-fokI, respectively. (**C**) Cell proliferation of U2OS cells expressing the indicated proteins with light exposure for 2 h at indicated recovery time. (**D**) Clonogenic survival of U2OS cells expressing KR-TRF1, DsR-TRF1 and NLS-KR after the indicated dose of light treatment. (**E**) Clonogenic survival of HeLa cells expressing DsR-TRF1 and KR-TRF1 after the indicated dose of light treatment. (**F**) MTT assay of IMR90 cells expressing KR-TRF1, DsR-TRF1 and NLS-KR after the indicated dose of light treatment. (**G**) SA-β-gal staining of DsR-TRF1 and KR-TRF1 stable-expressing HeLa cells. Percentages of SA-β-gal-positive cells (*n* = 100) are obtained from three independent experiments. Error bars are derived from SDs in three independent experiments from (a–e).

To further identify repair factors responsible for telomeric DNA damage repair at the single cell level, we analyzed the recruitment of base excision repair (BER) proteins after light induction, either throughout a single cell nucleus or at selected numbers of telomere spots (Supplementary Figure S2a–e). We found that the glycosylase NTH1, Polβ and FEN1 were recruited to activated KR-TRF1 or KR-TRF2 foci but not to DsR-TRF1, suggesting their specific enrichment at the sites of DNA damage at telomeres. We also observed that 53BP1, which is involved in DNA DSB repair (6), was localized at KR-TRF1 only after light illumination. To understand the similarity and differences between KR-TRF1 induced damage and enzyme-induced damage at telomeres, we compared the damage response of repair proteins at KR-TRF1 induced damage sites versus TRF1-Fok1 [which induces DSBs at telomeres (16)]. As shown in Figure 4a, DNA glycosylase NTH1 is not recruited to sites of TRF1-fok1 but to KR-TRF1. In contrast, γH2AX and 53BP1 are observed both at sites of TRF1-fok1 and KR-TRF1 after transfection and activation, respectively (Figure 4b, Supplementary Figure S2f). Therefore, damage induced by KillerRed specifically induces the BER pathway compared to an enzyme that induces double-strand DNA breaks. The recruitment of both Polβ and FEN1 to KR-TRF1 induced damage sites decreased (95 to 40%) after 24 h of recovery and a further decrease to <15% was seen 48 h after treatment (Supplementary Figure S2e). The dissociation of repair factors is likely indicative of repair completion (4). It is worth noting that KR-TRF1 utilizes ROS generated by the light activating chromophore to induce clustered oxidative damage (17). Clustered oxidative damage caused by ionizing radiation (IR) or the KR fusion proteins will lead to the production of DSBs in addition to base damage, and repair of DSBs at telomeres induced by nuclease has been explored in U2OS cells in a previous study (16). Our results indicate that BER plays an important role in the repair of both telomeric oxidative damage and chromosomal oxidative damage.

### Oxidative DNA damage at telomeres leads to cell death and cell senescence

Telomeric damage induced by KR-TRF1 leads to cell cycle arrest in Flp-in TREX 293 cells stably expressing KR-TRF1 with the induction of tetracycline and light (Supplementary Figure S3a). We observed an increased S phase population from 18 to 56.5% (Supplementary Figure S3b, red arrow) in KR-TRF1 cells 10 h after light activation, followed by a G2/M block at 16 h (Supplementary Figure S3b, blue arrow). In contrast, IR (2 Gy) treatment resulted in primarily a G2/M arrest (Supplementary Figure S3b, green arrow). These results suggest that the introduction of telomeric oxidative damage is sufficient to affect S phase progression. The delayed G2/M accumulation is presumably caused by secondary damage conversion into DSBs, which subsequently triggers the G2/M checkpoint.

We next examined the effect of KR-TRF1-induced telomeric damage on cell proliferation in U2OS cells expressing KR-TRF1 and DsR-TRF1 by continuous monitoring of cell growth following light activation (Figure 4c). Cells expressing KR-TRF1 without light activation showed a similar proliferation rate as cells expressing DsR-TRF1 (Figure 4c), suggesting that the impact of overexpressing KR-TRF1 on cell proliferation is similar to that of DsR-TRF1. Importantly, the cell proliferation rate exhibited the most profound decrease in cells expressing KR-TRF1 after damage induction (Figure 4c). We further examined if telomere-specific damage also affects cell survival using the colony formation assay (Figure 4d). U2OS cells expressing KR-TRF1 showed a dramatically diminished clonogenicity after light activation in a dose dependent manner. While DsR-TRF1 expressing U2OS cells had no apparent loss in cell survival when compared to control U2OS cells, only 5% of KR-TRF1 expressing U2OS cells survived after damage production with a light exposure of 4 h (Figure 4d).

We also determined cell survival in a HeLa cell line expressing active telomerase and a human diploid fibroblast IMR90 cell line without active telomerase. Increased cell loss was observed in KR-TRF1 but not DsR-TRF1 expressing HeLa cells (Figure 4e). Similarly, a decreased clonogenicity was also observed in the KR-TRF1 expressing IMR90 cell line compared to DsR-TRF1 expressing IMR90 cell lines (Figure 4f). These results collectively suggest that telomeric DNA damage leads to severe consequences for cellular proliferation and survival.

To determine if DNA damage at telomeres accelerates cellular senescence, we measured the senescence-associated β-galactosidase (SA-β-gal) in KR-TRF1- and DsR-TRF1-expressing HeLa cells after light exposure. Cells were grown for 30 passages and exposed to cool fluorescent light regularly for 10 min at each passage. After 30 passages, KR-TRF1-expressing cells showed significantly higher SA-β-gal-positive cells compared to DsR-TRF1 expressing cells (Figure 4g) with identical light exposure, indicating that oxidative damage at telomeric sites is a strong inducer of cellular senescence.

### Telomere-specific DNA damage disrupts telomere integrity and accelerates telomere shortening

**Figure 5.**
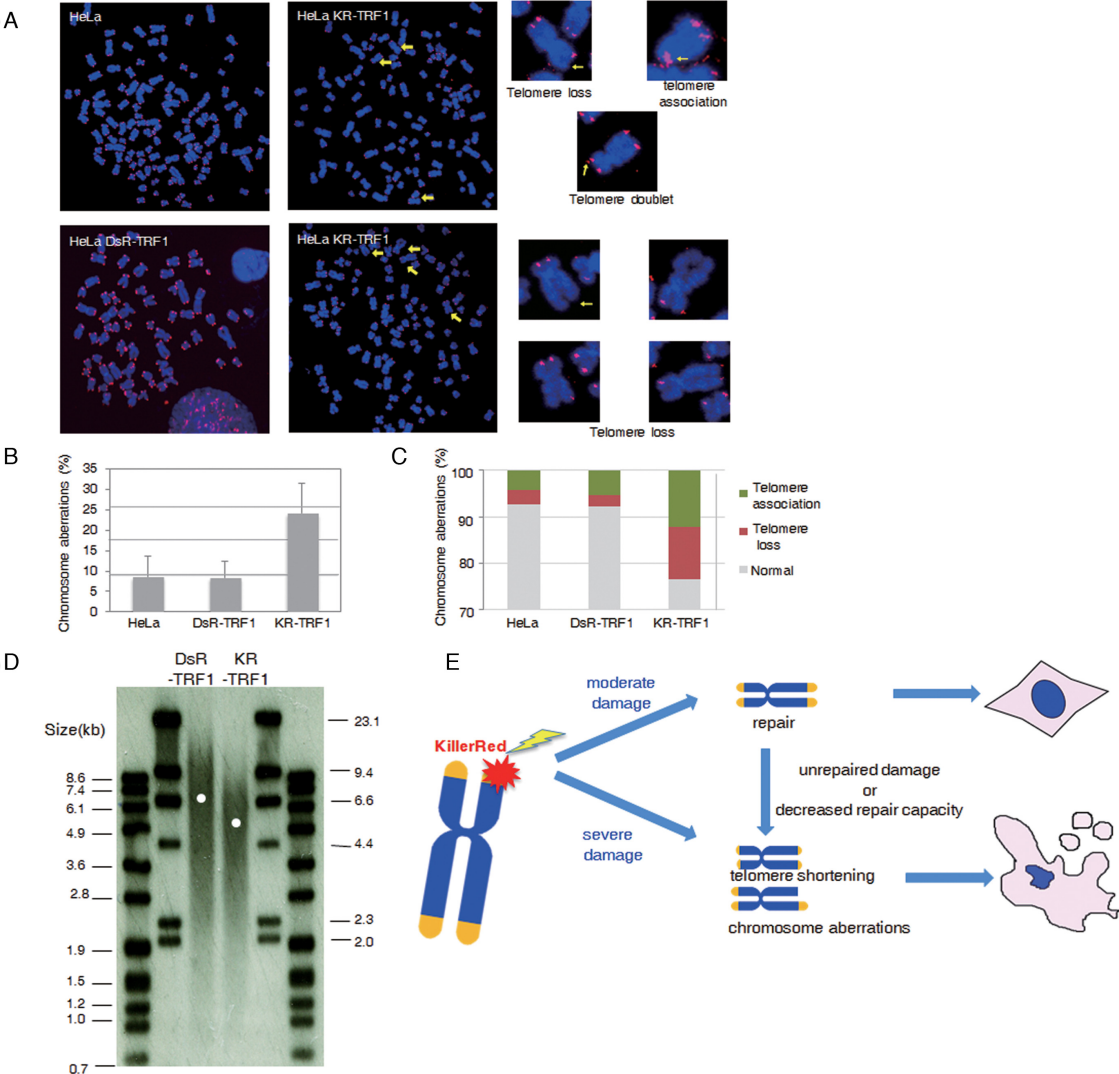
Telomere-specific DNA damage disrupts telomere integrity and accelerates telomere shortening. (**A**) Chromosomal aberrations found in HeLa cells expressing KR-TRF1. (**B** and **C**) Quantification of chromosomal aberrations from (a). Aberrations per chromosome (b) and percentage of each chromosomal aberration type (c) are shown. Data were derived from three independent experiments (*n* = 2000). (**D**) TRF analysis of average telomere length in HeLa cells. (**E**) A model depicting oxidative DNA damage at telomeres leading to cell death. Abbreviation lists: KR-TEL, KillerRed induced DNA Damage at Telomeres; ROS, reactive oxygen species; KR, KillerRed; tetR, tetracycline repressor; TA, transcription activation; TRE, tetracycline responsive element; PNA, peptide-nucleic acid; BER, base excision repair; PARP, Poly (ADP-ribose) polymerase; PAR, poly ADP-ribosylation; SSBs, single strand breaks; DSBs, double strand breaks; DDR, DNA damage response; oxo-G, 8-oxo-guanine; TRF1, telomere repeat binding factor 1; TRF2, telomere repeat binding factor 2; high LET, high linear energy transfer.

Next, we investigated the nature of telomere dysfunction arising from telomere-specific DNA damage. Previously, H_2_O_2_ exposure has been shown to induce chromosome fragmentation which is considered the major type of chromosomal aberration (27,28). The specific impact on telomeres, however, was most likely masked by the low proportion of telomere sequences within the genome. To determine the nature of chromosomal aberrations induced by telomeric oxidative damage, we analyzed the chromosomal aberrations in HeLa cells stably expressing DsR-TRF1 or KR-TRF1 after light activation. As shown (Figure 5a), light induction led to a marked increase in chromosomal aberrations from 8% in DsR-TRF1 expressing cells to 24% in KR-TRF1 expressing cells. We found that telomeric damage mainly led to telomere associations and chromatid telomere loss (Figure 5a and b); both types of chromosomal aberration were rarely observed in either IR- or H_2_O_2_-treated cells (27,28). We also found other types of chromosomal aberration, including cruciform chromosomes and telomere associations, which were undetectable in wild-type (WT) or DsR-TRF1 expressing cells, but exist at 1% frequency in KR-TRF1 treated HeLa cells (Figure 5c). Additionally, examination of the terminal restriction fragments showed that HeLa cells stably expressing KR-TRF1 or DsR-TRF1 after damage induction exhibited an average shortening of ∼2 kb in KR-TRF1 cells compared to DsR-TRF1 cells (Figure 5d). This result clearly indicates that oxidative DNA damage at sites of telomeres leads to accelerated telomere erosion that gives rise to chromosomal abnormalities.

## DISCUSSION

The KR-TRF1 fusion protein, which is targeted specifically to the telomeres by the shelterin protein (2), functions as both a ‘label’ and ‘a remotely controlled bomb’ at telomeric sites in live cells (Figure 1). Using KR-TRF1 to analyze the DNA damage response at telomeres has several advantages. First, KR-TRF1 precludes the global cellular effects of non-telomeric DNA damage. Second, incorporation of KR-TRF1 does not interfere with the natural telomeric structure. Third, KillerRed induces oxidative DNA damage which can be quantitatively modulated by light exposure dosage. Fourth, KR can be activated in real time at an individual telomere in live cells. Compared to telomeric damage induced by site-specific endonucleases (16), in which the DNA damage response at telomeres is hard to follow in the short-term due to minimal damage production by endonuclease at telomeres, the damage response process at telomeres induced by KR-TRF1 can be observed from damage production through repair completion in real time after KR activation. These advantages make it possible to analyze the precise mechanisms of telomere protection. Using a novel method, we have shown definitively that the repair of oxidative lesions accelerates telomere stability and chromosomal integrity.

Previous studies suggested that DSBs are irreparable at telomeres after global irradiation of cells (29). However, our results clearly show that DSBs at more than half of the telomeres are repaired at 48 h after light illumination. This discrepancy may be explained by different damage induction strategies. The previous study used whole cell irradiation and detected unrepaired DSBs at telomeres (the average number was ∼5 foci per nucleus) at 72 h after irradiation, while we induced DNA damage specifically at telomeres and followed their repair at the individual telomere level. The efficient recruitment of DNA repair factors and subsequent dissociation kinetics indicate that the repair has occurred at sites of telomeres (Figures 2 and 3). Interestingly, the average number of unrepaired telomeres is very similar (the average number is ∼5 foci per nucleus in our assay). However, with global damage approaches, exceedingly high doses of exogenous chemical exposure or radiation are needed in order to inflict a sufficient amount of telomere damage. The global impact and the potential repair capacity limitations could hamper the cell's ability to repair telomere DNA damage. Therefore, our observation was obtained in more physiologically relevant conditions that are less influenced by factors such as global cellular stress or capacity saturation. Our results certainly support the idea that telomeres are sensitive to damage caused by IR and this is associated with replicative senescence (29,30), but further demonstrate the repair of damage at single telomere levels.

Our system introduces an extensive amount of localized oxidative damage that might result in a high frequency of multiply damaged sites, i.e. various types of damage. It is known that multiple damage, including base damage, single strand and DSBs, poses problems for repair, and such sites are repaired slowly (31). Therefore, KR-induced damage mimics damage induced by IR, especially high linear energy transfer (high LET) radiation (32,33). Previous studies used IR to induce damage throughout the cell nucleus and showed that DSBs are irreparable at telomeres after global IR of cells (29). The retention of γH2AX shown in our study (Figure 3) supports the notion that repair of DSBs or multiply damaged sites at telomeres is also slow at some telomeres, i.e. longer ones. More importantly, clustered DNA lesions have greater cell killing effectiveness (33) and given that KillerRed has the potential to induce such multiple lesions locally in a dose-dependent manner, our method should permit the analysis of repair mechanisms at multiply damaged sites. Importantly, while our method may produce multiple types of DNA damage, the current work is the first to focus specifically on damage at telomeres, recognizing that naturally occurring intracellular oxidative damage does not produce multiple types of lesions. The delayed repair of 8-oxoG and strand breaks at long telomeres in U2OS cells can also be attributed partly to the initial occurrence of damage, presumably proportional to the extent of TRF1 occupancy at the telomeres. Mice lacking Ogg1 glycoslyase, which removes oxidized bases, exhibit telomere aberrations (34). This suggests that telomere stability requires the repair of oxidized bases at telomeres and previous biochemical studies show that telomere binding proteins interact with many BER proteins and stimulate their activities *in vitro* (35,36). Compared to the delayed repair of 8-oxoG at long telomeres in U2OS cells, we did not observe an apparently enhanced portion of unrepaired 1–4 oxoG foci at KR-TRF1 sites in HeLa cells with homogenous, short telomeres (Supplementary Figure S1f), supporting the notion that the inefficient repair of long telomeres in U2OS cells is more likely due to the inefficient processing by repair proteins within the telomere structure. Therefore, it is also conceivable that longer telomeres are intrinsically more difficult for repair factor access or local availability, given the more complex or unique structures associated with longer repeats.

We have measured cell viability curves in U2OS, HeLa and IMR90 cells. Under the condition of stably expressing KR-TRF1, cells from telomerase-negative lines, i.e. U2OS and IMR90, are more sensitive to telomeric DNA damage than telomerase-active HeLa cells. In U2OS and IMR90 cells, a relatively lesser amount (1.5–3 h) of light illumination is needed to reach the IC50 for cell death than is the case for the exposure (>5 h) in HeLa cells, showing that the HeLa cell line is more resistant to damage than U2OS and IMR90. These results indicate that: (i) U2OS cells are more vulnerable to death due to a dependency on recombination and repair for maintaining telomere length and therefore, they are more sensitive to oxidative damage and (ii) telomerase might contribute to long-term cell survival under oxidative stress. Future studies to investigate the role of telomerase in the face of oxidative stress-induced telomere shortening are planned.

In summary, our study offers the first evidence that telomeric DNA damage *per se* is sufficient to induce cell senescence and cell proliferation arrest (Figure 4). We also identified that the major types of chromosomal aberration induced by telomeric damage are chromatid telomere loss and telomere associations. These are likely signature chromosomal aberrations associated with oxidative telomere damage. The high frequencies of chromatid telomere loss and telomere associations are fully consistent with telomere defects caused by telomeric oxidative damage. They are also distinct from the chromosomal aberrations observed with IR or H_2_O_2_ treatment. In addition, although telomeric damage induces telomere loss, it does not appear that non-homologous end joining (NHEJ) is substantially activated since activated NHEJ at telomeres will induce chromosome fusion as was observed with TRF2 suppression (37). Therefore, telomeric DNA damage might not primarily activate NHEJ. Global genome damage has been found to induce aberrations such as fragmentation, fusion and dicentric chromosomes (27,28). In addition, sensitivity to DNA DSBs has been shown to be associated with an increased frequency of large deletions and chromosome rearrangements (38,39). These aberrations, while consistent with chromosomal breakage formation, may have masked the telomeric impact of oxidative DNA damage.

We showed that oxidative DNA damage at telomeres leads to the strong recruitment of BER factors, suggesting that BER is the primary mechanism for oxidative damage repair, similar to chromosomal oxidative damage. When the amount of oxidative damage is moderate, repair is expected to be at high efficiency. Clustered oxidative damage caused by ionizing radiation or the KR fusion proteins will produce DSBs in addition to single-strand breaks and base damage. Therefore, when cells experience severe clustered oxidative damage, it may result in gross chromatid telomere loss and consequently telomere associations (Figure 5e). Our observation that oxidative telomere damage leads to telomere shortening may provide another clue as to how oxidative damage, a constant endogenous threat, may contribute significantly to telomeric erosion. Combined with the reduced repair capacity associated with ageing, such erosion may be further accelerated and eventually lead to pathological consequences. Future studies based on our finding and our new technical platform may facilitate the development of new therapeutic strategies for better preserving telomere integrity during aging or strategies to efficiently kill cancer cells by targeting the unique mechanism through which cancer cells maintain telomere stability.

## SUPPLEMENTARY DATA

Supplementary Data are available at NAR Online.

SUPPLEMENTARY DATA
