# Supplementary material for: Targeted DNA damage at individual telomeres disrupts their integrity and triggers cell death
Source: Nucleic Acids Res. 2015 Jun 15;43(13):6334–47. doi: 10.1093/nar/gkv598 (PMC4513870; doi:10.1093/nar/gkv598)
Supplement: SUPPLEMENTARY DATA [file supp_43_13_6334__index.html]

Targeted DNA damage at individual telomeres disrupts their integrity and triggers cell death — SUPPLEMENTARY DATA 

# Targeted DNA damage at individual telomeres disrupts their integrity and triggers cell death

## SUPPLEMENTARY DATA

- SUPPLEMENTARY DATA
- SUPPLEMENTARY DATA
