# Supplementary material for: Targeted DNA damage at individual telomeres disrupts their integrity and triggers cell death
Source: Nucleic Acids Res. 2015 Jun 15;43(13):6334–47. doi: 10.1093/nar/gkv598 (PMC4513870; doi:10.1093/nar/gkv598)

**Supplementary Figure 1. a)** Co-labeling of telomeres using KR-TRF1 and sgTelomere$^{(F+E)}$-directed dCas9-EGFP. **b)** Expression of KR-TRF1 in indicated cell lines. **c)** Expression of DsR-TRF1, KR-TRF1 and NLS-KR in U2OS cells. **d)** Stable expression of DsR-TRF1 and KR-TRF1 in HeLa cells used in Fig. 4 and Fig. 5. **e)** 8-oxoG staining in KR-TRF1 or DsR-TRF1 expressing HeLa 1.3 cells with light activation for 20 min. **f)** Kinetics of 8-oxoG at KR-TRF1 sites in transfected HeLa cells with or without light activation of KR, recovered in the dark for the indicated time.

**Supplementary Figure 2. Kinetics of DNA repair factor recruitment at telomeric site-specific damage. a-d)** Recruitment of NTH1 (*a*), Polβ (*b*), FEN1 (*c*) and 53BP1 (*d*) to damage sites of KR-TRF1 at a single telomere spot in a single cell nucleus, or selected numbers of telomere spots as indicated with a yellow rectangle. **e)** Quantification of the percentage of cells showing co-localization with KR-TRF1 and Polβ or FEN1, with the indicated recovery time after 20 min of 15 W Sylvania cool white fluorescent bulb activation of KR. Mean values with a SD for 150 cells are given. P-value is calculated by Student's t-test, ** $P<0.005$. **f)** Staining of γH2AX (green) at sites of Flag-TRF1-fokI (red).

**Supplementary Figure 3. Telomeric oxidative DNA damage induces cell cycle progression defects. a)** Cell-cycle profiles of Flp-in T-REX 293 cells stably expressing KR-TRF1 with tetracycline induction. $1x10^5$ cells were seeded in 60 mm culture dishes and treated with cool fluorescent light for 2 hr, followed by the indicated recovery time in the dark. DNA contents were measured by propidium iodide staining for flow cytometry. The percentages of each cell-cycle phase at the indicated recovery time are shown. **b)** Percentage of cells in G1, S and G2/M phase was quantified in each cell after treatment as in (*a*).

**Supplementary video**

**Three dimensional image of KR-TRF1 (red) and DAPI (blue) in a U2OS cell nucleus by SIM.** The 3D-SIM microscopy system was employed to carry out the 3D super-resolution imaging of U2OS cells. U2OS cells expressing KR-TRF1 were fixed with 3.7% (v/v) formaldehyde. The wavelengths of 405 nm and 561 nm were used to excite DAPI (blue) and KR-TRF1 (red) in the cell nucleus, respectively. The lateral resolution of the 3D-SIM image is ~100 nm and the axial resolution is ~240 nm.

**Supplementary Figure 1**

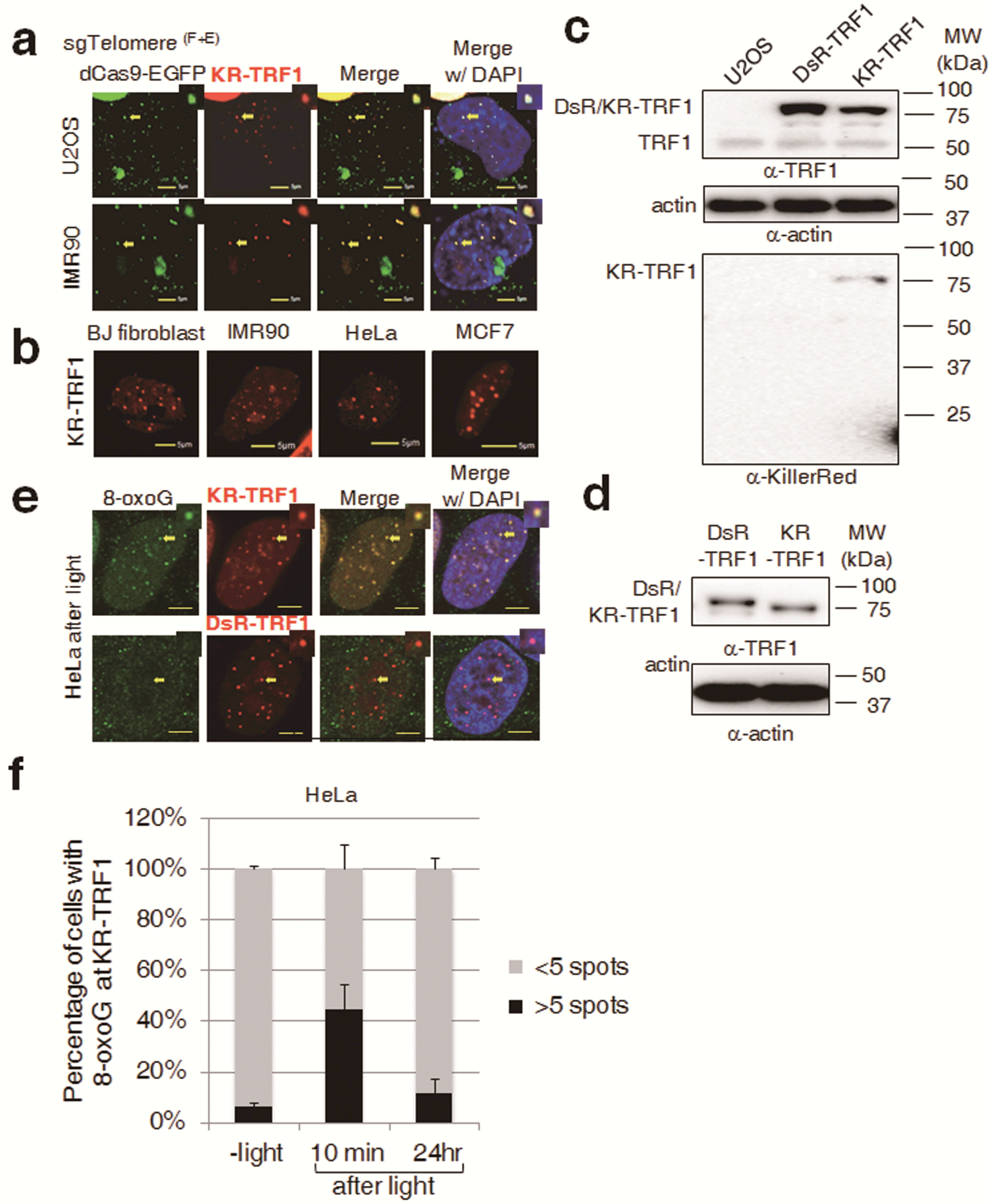

**Supplementary Figure 2**

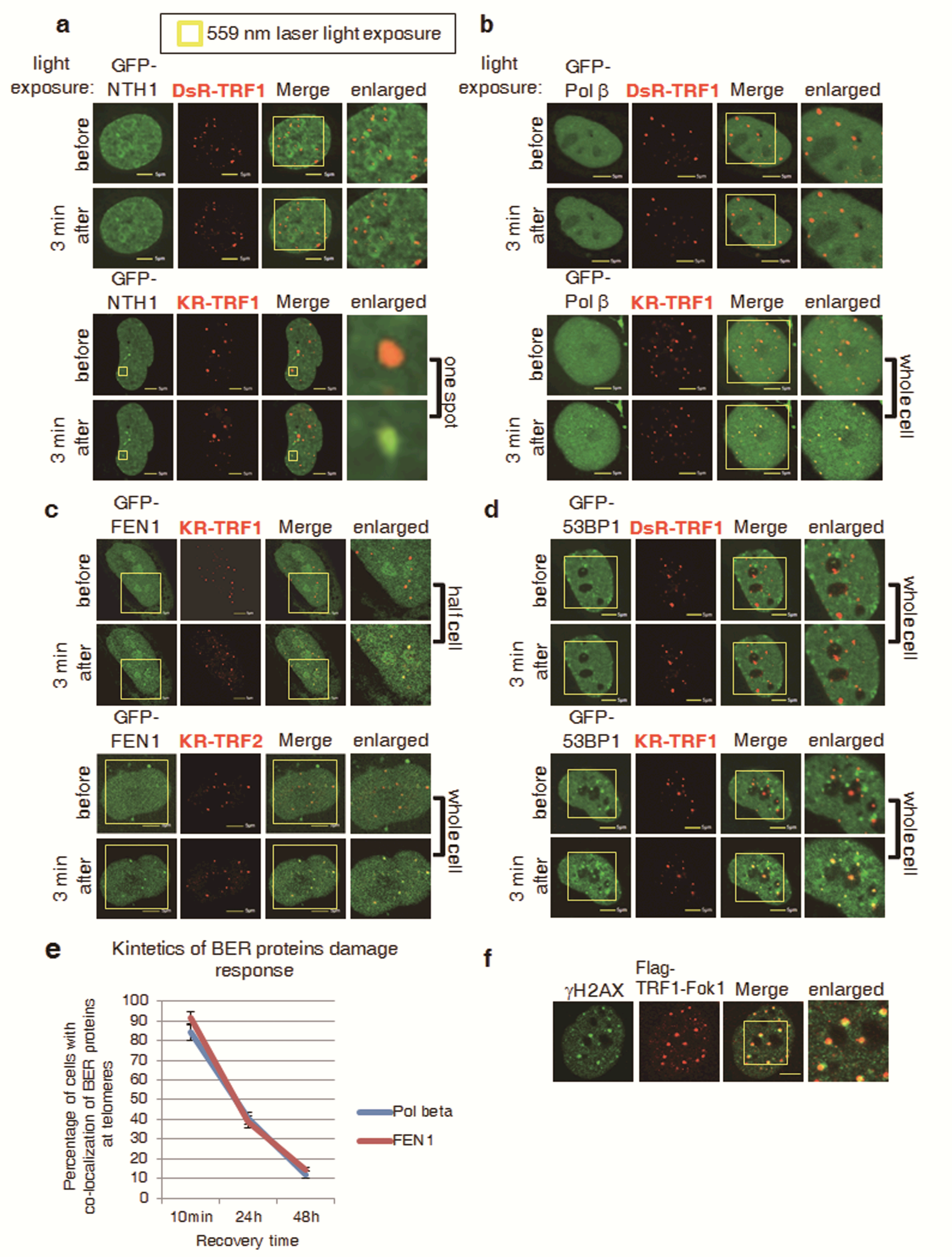

**Supplementary Figure 3**

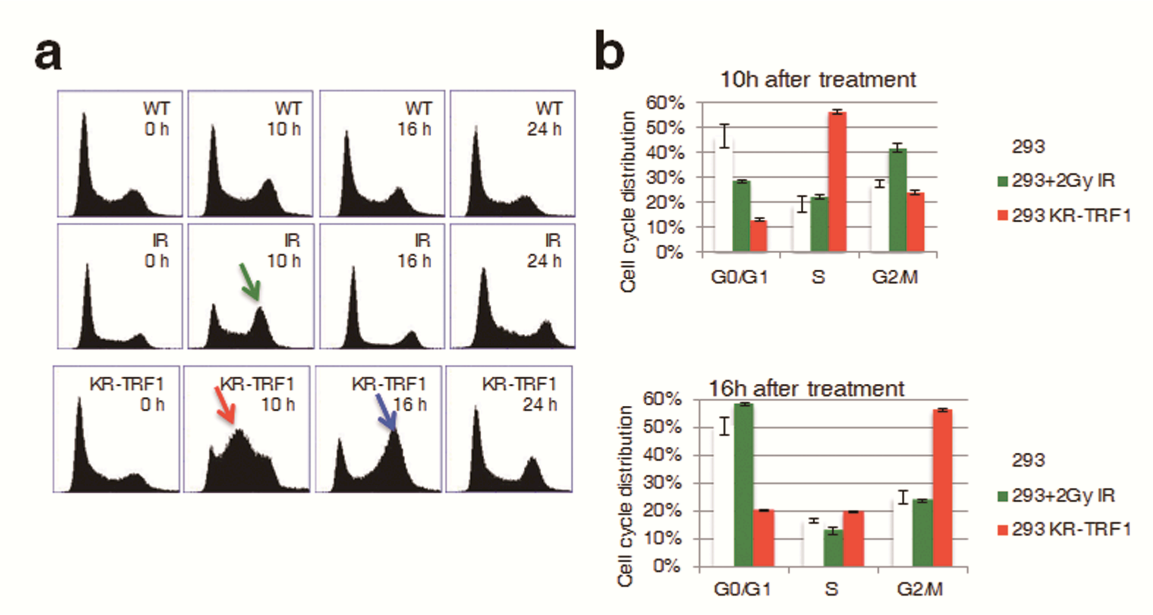

Supplement: SUPPLEMENTARY DATA [file supp_gkv598_nar-01269-d-2015-File007.pdf]
